# miR-499-5p Attenuates Mitochondrial Fission and Cell Apoptosis via p21 in Doxorubicin Cardiotoxicity

**DOI:** 10.3389/fgene.2018.00734

**Published:** 2019-01-21

**Authors:** Qinggong Wan, Tao Xu, Wei Ding, Xuejuan Zhang, Xiaoyu Ji, Tao Yu, Wanpeng Yu, Zhijuan Lin, Jianxun Wang

**Affiliations:** ^1^Center for Regenerative Medicine, Institute for Translational Medicine, College of Medicine, Qingdao University, Qingdao, China; ^2^School of Basic Medical Sciences, Qingdao University, Qingdao, China; ^3^Department of Comprehensive Internal Medicine, Affiliated Hospital, Qingdao University, Qingdao, China

**Keywords:** doxorubicin, miR-499-5p, p21, cardiotoxicity, mitochondrial, apoptosis

## Abstract

Doxorubicin (DOX) is a broad-spectrum anti-tumor drug, but its cardiotoxicity limits its clinical application. A better understanding of the molecular mechanisms underlying DOX cardiotoxicity will benefit clinical practice and remedy heart failure. Our present study observed that DOX caused cardiomyocyte (H9c2) apoptosis via the induction of abnormal mitochondrial fission. Notably, the expression levels of p21 increased in DOX-treated cardiomyocytes, and the silencing of p21 using siRNA greatly attenuated mitochondrial fission and apoptosis in cardiomyocytes. We also found that miR-499-5p could directly target p21 and attenuated DOX-induced mitochondrial fission and apoptosis. The role of the miR-499-5p-p21 axis in the prevention of DOX cardiotoxicity was also validated in the mice model. DOX treatment induced an upregulation of p21, which induced subsequent abnormal mitochondrial fission and myocardial apoptosis in mouse heart. Adenovirus-harboring miR-499-5p-overexpressing mice exhibited significantly reduced p21 expression, mitochondrial fission and myocardial apoptosis in hearts following DOX administration. The miR-499-5p-overexpressing mice also exhibited improved cardiomyocyte hypertrophy and cardiac function after DOX treatment. However, miR-499-5p was not involved in the DOX-induced apoptosis of cancer cells. Taken together, these findings reveal an emerging role of p21 in the regulation of mitochondrial fission program. miR-499-5p attenuated mitochondrial fission and DOX cardiotoxicity via the targeting of p21. These results provide new evidence for the miR-499-5p-p21 axis in the attenuation of DOX cardiotoxicity. The development of new therapeutic strategies based on the miR-499-5p-p21 axis is a promising path to overcome DOX cardiotoxicity as a chemotherapy for cancer treatment.

## Introduction

Doxorubicin (DOX), also known as adriamycin (ADR), exerts a killing effect on a variety of tumors, and it is a widely used anti-tumor drug (Levis et al., 2017). However, a previous study found that DOX produced heart damage in many patients that eventually led to heart failure (Gorini et al., 2018). The development of treatment strategies that avoid DOX cardiotoxicity without affecting its anti-tumor effects is urgently needed. A previous study found that DOX-induced cardiotoxicity primarily involved the production of reactive oxygen species (ROS) (Ichikawa et al., 2014), lipid peroxidation (Yarana et al., 2018), DNA damage (Li et al., 2009), mitochondrial dysfunction (Dhingra et al., 2014), apoptosis (Kumar et al., 2001), and autophagy dysregulation (Bartlett et al., 2017). These toxicities ultimately caused cell death of cardiomyocyte. Therefore, a better understanding of the molecular mechanisms underlying DOX cardiotoxicity will improve the clinical application of DOX during cancer therapy (Kluza et al., 2004).

MicroRNAs (miRNAs) are endogenous non-coding RNAs that are highly conserved in different species (Lim et al., 2003). miRNAs functionally participate in the developmental, physiological and pathological process via negative regulation of target gene (Bartel, 2009; Chistiakov et al., 2016). Increasing evidence suggests that miRNAs are actively involved in the regulation of cardiac functions, such as electrical signal conductance, heart muscle contraction, heart growth and morphogenesis (Kozomara and Griffiths-Jones, 2011). What’s more, miRNAs are also powerful regulators of cardiovascular diseases, and manipulation of miRNAs is a promising strategy for the development of novel therapeutic agents (Mette et al., 2002; Liu S. et al., 2018; Tang et al., 2018).

miR-499-5p is a recently discovered member of myosin-encoded miRNAs (Olivieri et al., 2013). Several studies reported that miR-499-5p was differentially regulated and functioned in the heart development process (Sluijter et al., 2010; Wilson et al., 2010; Fu et al., 2011; Wander et al., 2016). miR-499-5p is expressed at a high level in the heart under physiological conditions, and it attenuates the expression of the β-myosin heavy chain, which results in enhanced myocardial oxygen metabolism and tolerance. miR-499-5p is downregulated in human heart diseases and experimental models of heart failure, and it is involved in the transcriptional and posttranslational regulation of pathological hypertrophy (Chistiakov et al., 2016). Several lines of evidence suggest that miR-499-5p exerts cardioprotective effects via the protection of cardiomyocytes from stress-induced apoptosis. miR-499-5p inhibits calcineurin-mediated dephosphorylation of dynamin-related protein-1 (Drp1) via the targeting of CnA α and CnA β, which reduces Drp-1 mitochondrial aggregation and attenuates Drp-1-mediated mitochondrial fission (Wang et al., 2011). miR-499-5p also targets several regulatory factors that inhibit mitochondrial cell apoptosis and increase cell survival, such as bispecific tyrosine phosphorylation regulatory kinase 2 (Dyrk2), programmed cell death protein 4 (Pdcd4) (Li Y. et al., 2016) and phosphoric acid Forint acid cluster sorting protein 2 (Pasc2) (Wang J. et al., 2014). However, the potential effects and underlying mechanisms of miR-499-5p in the protection against DOX-induced heart failure have not been revealed.

Cyclin-dependent kinase inhibitor 1a (CDKN1a), also known as p21, is a negative regulator that halts cell cycle progression at the G1/S and G2/M transition points via inhibition of CDK4,6/cyclin-D and CDK2/cyclin-E, respectively (Sharpless and Sherr, 2015). Mammalian p21 is expressed at very low levels in embryonic and neonatal hearts (Brooks et al., 1998). Numerous studies demonstrated that p21 was involved in the pathological process of myocardial injury (Wang et al., 2015a). Inhibition of p21 prevented endothelial cell apoptosis in arsenite-induced endothelial dysfunction-related vascular diseases (Nuntharatanapong et al., 2005). Increased p21 levels in cardiac fibroblasts surrounding an infarction area is a biomarker of hyperoxia perception (Sen et al., 2006). Hyperoxia causes cardiac remodeling via the induction of p21-dependent differentiation of cardiac fibroblasts (Roy et al., 2003a,b). Abnormal p21 expression is closely related to myocardial damage and cardiac hypertrophy (Wang R. et al., 2014). Transverse aortic constriction (TAC) surgery and DOX administration concomitantly increased p21 levels in rat and mouse cardiomyocytes (Terrand et al., 2011; Koga et al., 2013). Increased p21 expression in tissues mediates myocardial fibrosis and remodeling (Kuhn et al., 2007; Megyesi et al., 2015). However, the mechanism of p21 regulation of myocardial damage and the regulation of the p21 expression in cardiomyocytes under stress have not been elucidated.

Our present study investigated the molecular mechanisms underlying DOX cardiotoxicity. We found an emerging role of p21 in promoting mitochondrial fission and cardiomyocyte apoptosis via regulation of mitochondrial fission programming induced by DOX treatment. miR-499-5p inhibited DOX-induced mitochondrial fission and apoptosis in cardiomyocytes via the targeting of p21. DOX cardiotoxicity was prevented in adenovirus-harboring miR-499-5p-overexpressing mice, and miR-499-5p overexpression improved cardiac function. Our research demonstrated that the miR-499-5p-p21 axis constitutes a new antiapoptotic pathway to attenuate DOX-induced cardiotoxicity and may provide valuable insights to prevent DOX cardiotoxicity during cancer chemotherapy.

## Materials and Methods

### Cell Culture and Treatment

H9c2 cells were purchased from the Shanghai Institutes for Biological Sciences (Shanghai, China). The human lung cancer cell line A-549, the human gastric cancer cell line SGC-7901, the human hepatocellular carcinoma cell line HepG-2 and the human colorectal cancer cell line SW-480 were purchased from the Chinese Academy of Sciences Cell Bank. Cells were maintained in Dulbecco’s modified Eagle’s medium (DMEM) supplemented with 10% fetal bovine serum (FBS) and antibiotics (100 IU/mL penicillin and 100 mg/mL streptomycin) in a humidified atmosphere of 5% CO2 at 37°C. Cells were treated with 2 or 0.2 μM DOX, unless indicated otherwise.

### Animal Experiments

Mice (C57BL/6, male, 7 weeks old) were injected with DOX or saline. Briefly, mice were treated twice per week with 10 mg/kg DOX or control saline solution for 1 week (20 mg/kg cumulative dose of DOX). Heart tissues were analyzed 1 week after the last treatment. DOX doses were based on previous reports (Zhu et al., 2009). Cardiac function and ventricular remodeling were investigated 1 week after the last treatment. All procedures involving animals were reviewed and approved by the Institutional Animal Care and Use Committee of Qingdao University Medical College.

### Quantitative Real-Time PCR (qRT-PCR)

Stem-loop qRT-PCR was performed in an Applied Biosystems ABI Prism 7000 sequence detection system. Total RNA was extracted using TRIzol reagent. DNase I (Takara, Otsu, Japan) was applied, and RNA was reverse-transcribed using a reverse transcriptase kit (Takara). Mature miR-499-5p levels were measured using SYBR Green Real-time PCR Master Mix (Takara) according to the manufacturer’s instructions. The same reverse primer with the sequence 5′-GTGCAGGGTCCGAGGT-3′ was used for all miRNAs. The primer used is as described in Table 1.

**Table 1:** The primer used in this study.

miR-499-5p	Forward: 5′-GGTCCAGACTGGGGTCCCAGC-3′ Reverse: 5′-GCATGCCGCAGTGGTTAGGGA-3′
U6	Forward: 5′-GCTTCGGCAGCACATATACTAA-3′ Reverse: 5′-AACGCTTCACGAATTTGCGT-3′
ANP	Forward:5′-TCCGATAGATCTGCCCTCTTGAA-3′ Reverse:5′-GTACCGGAAGCTGTTGCAGCCTA-3′
β-MHC	Forward: 5′-CAGACATAGAGACCTACCTTC-3′ Reverse: 5′-CAGCATGTCTAGAAGCTCAGG-3′
GAPDH	Forward: 5′-TGGAGTCTACTGGCGTCTT-3′ Reverse: 5′-TGTCATATTTCTCGTGGTTCA-3′
miR-16	Forward: 5′-TAGCAGCACGTAAATATTGGCG-3′ Reverse: 5′-GTGCAGGGTCCGAGGT-3′
p21 3′UTR	Forward:5′-ATGATATCCTTCTGCTGTGGGTCA-3′ Reverse:5′-GGACTAGTATTGCACGAGGGGAG-3′

### Mitochondrial Staining

We performed mitochondrial staining as described previously (Boman et al., 2000; Wang et al., 2015b). Briefly, we plated cells onto coverslips. Cells were stained after treatment for 20 min with 0.02 μM MitoTracker Red CMXRos (40743ES50; Yeasern, Shanghai, China). We imaged mitochondria using a laser scanning confocal microscope (Zeiss LSM510 META, Jena, Germany). We randomly measured at least 150 cells from each experiment to determine the percentage of cells undergoing mitochondrial fission.

### Immunoblotting

Immunoblotting was performed to determine the expression levels of p21 and actin. Briefly, cells were lysed for 1 h at 4°C in lysis buffer (20 mM Tris pH 7.5, 2 mM EDTA, 3 mM EGTA, 2 mM DTT, 250 mM sucrose, 0.1 mM phenylmethylsulfonyl fluoride, and 1% Triton X-100) containing a protease inhibitor cocktail. Protein samples were subjected to 12% SDS-PAGE and transferred to nitrocellulose membranes. Blots were probed using corresponding primary antibodies. Horseradish peroxidase-conjugated secondary antibodies were used. Antigen-antibody complexes were visualized using enhanced chemiluminescence. Equal protein loading was controlled using Ponceau Red staining of membranes. Antigen-antibody complexes were visualized using enhanced chemiluminescence. Bands were quantitated using Image J and α-actin was used as the loading control. Fold change was normalized to the indicated control. Information on the antibodies used is described in Table 2.

**Table 2:** The information of the antibodies used in his work.

Antibody	Source	Dilutions	Company
p21	Rabbit	1:1000	Wanleibio, China
β-action	Mouse	1:5000	Sungenebio, China
Anti-Mouse IgG	Rabbit	1:5000	Biovision, United States
Anti-Rabbit IgG	Goat	1:5000	Biovision, United States

### Cell Death Assay

Cell death was determined using a trypan blue exclusion assay. Trypan blue-positive and trypan blue-negative cells were counted using a hemocytometer (Liu et al., 2009). We randomly measured 150 cells from each experiment to calculate the cell death rate.

### Apoptosis Assays and Histology

Apoptosis was determined using terminal deoxyribonucleotidyl transferase-mediated TdT-mediated dUTP nick-end labeling using a kit from Yeasern (Alexa Fluor 488). Harvested hearts were fixed in 4% paraformaldehyde, embedded in paraffin and sectioned at a 6-μm thickness. Detection procedures were performed in accordance with the kit instructions (He et al., 2017). We randomly measured 150 cells from each experiment to calculate the apoptotic rate.

### Echocardiographic Assessment

Echocardiography was performed as described previously. Generally, mice were mildly anesthetized, and transthoracic echocardiography was performed using a Vevo 2100 high-resolution system (VisualSonics, Toronto, ON, Canada). Two-dimensional guided M-mode tracings were recorded in parasternal long and short axis views at the level of the papillary muscles. Systolic left ventricular internal diameter (LVIDs) and diastolic left ventricular internal diameter (LVIDd) were measured. We calculated the fractional shortening (FS) of the left ventricular diameter as (LVIDd – LVIDs)/LVIDd] × 100. All measurements were obtained for greater than three beats and averaged. Mice were euthanized after *in vivo* evaluations of cardiac function, and hearts were harvested and weighted prior to histological examination (Coppola et al., 2016).

### Electron Microscopy

Heart ultrastructural analysis was performed to quantify mitochondrial fission. Sample preparations and conventional electron microscopy were performed as described (Cadete et al., 2016). Samples were examined at a magnification of 15,000 using a JEOL JEM-1230 transmission electron microscope. Electron microscopy micrographs of thin sections were evaluated for comparisons of mitochondrial fission. The sizes of individual mitochondria were measured using Image-Pro Plus software. We defined mitochondria smaller than 0.6 μm^2^ as fission mitochondria (Wang et al., 2015d).

### Reporter Construction and Luciferase Assay

The p21 3′UTR was amplified from mouse genomic DNA using PCR. The primers were as described in Table 1. PCR products were gel-purified and ligated into a pGL3 reporter vector (Promega) immediately downstream of the stop codon of the luciferase gene. Mutations of the p21 3′UTR construct were introduced using a QuikChange II XL site-directed mutagenesis kit (Stratagene). The p21 3′UTR-Mut (the wild-type p21 3′UTR site: AGUCUUAA, p21 3′UTR-Mut: AGACGGAA) was produced using a QuikChange II XL Site-Directed Mutagenesis Kit (Stratagene, La Jolla, CA, United States). A luciferase activity assay was performed as described previously (Wang et al., 2015c). Briefly, cells were cultured in 24-well plates, infected with miR-499-5p mimic or negative control and transfected with the plasmid construct pGL3-p21-3′UTR or pGL3-p21-3′UTR-Mut at a concentration of 200 ng/well using Lipofectamine 3000 (Invitrogen). The Renilla luciferase plasmid was cotransfected at 2.5 ng/well and served as the internal control. Cells were lysed 48 h after transfections, and luciferase activity was detected using a Dual Luciferase Reporter Assay kit (Promega). All experiments were performed in triplicate.

### Construction of Adenovirus and Overexpression Vector

miR-499-5p-overexpressing adenovirus and adenovirus β-galactosidase (β-gal) were prepared as described previously (Wang et al., 2011). All adenoviruses were amplified in HEK-293 cells. Adenoviral infection of cells was performed as described previously (Wang et al., 2009). The open reading frame (ORF) of the p21 gene was generated using RT-PCR, and p21 siRNA was purchased from Genepharma (Shanghai, China). P21 was cloned into the pcDNA3.1 expression vector according to the manufacturer’s guidelines (Invitrogen). The constructed sequence was further confirmed using sequencing.

### Data and Statistical Analysis

All values are expressed as the means ± standard error. *n* = 3. Statistical significance was defined as *p* < 0.05. One- or two-way analysis of variance (ANOVA) was used to test each variable for differences between treatment groups. If ANOVA demonstrated a significant effect, then pairwise *post hoc* comparisons were performed using Fisher’s least significant difference test.

## Results

### miR-499-5p Attenuates Mitochondrial Fission and Apoptosis in Cardiomyocytes Treated With DOX

**FIGURE 1:**
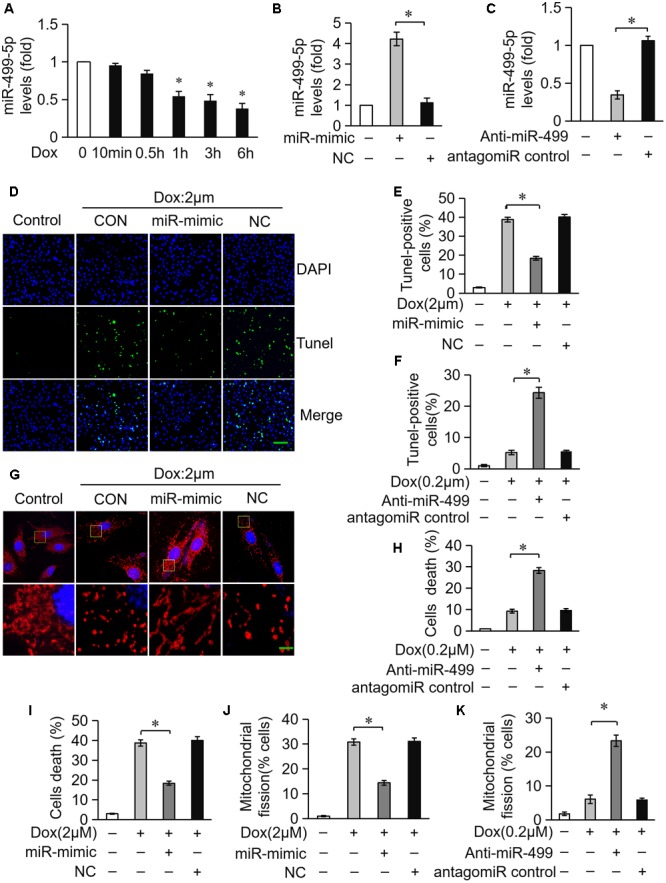
miR-499-5p attenuates mitochondrial fission and apoptosis in cardiomyocytes treated with DOX. **(A)** Measurement of miR-499-5p mRNA levels in H9c2 cells treated with 2 μM DOX at the indicated times. **(B)** Transfection with a miR-499-5p mimic forced the expression of miR-499-5p in cardiomyocytes. Cells were transfected with miR-499-5p mimic (miR-mimic) or negative control (NC) for 24 h. The expression levels of miR-499-5p were detected using real-time PCR. **(C)** miR-499-5p antagomiR knocked down endogenous miR-499-5p. Cells were transfected with a miR-499-5p antagomiR (Anti-miR-499) or antagomiR control for 24 h. The expression levels of miR-499-5p were detected using real-time PCR. **(D)** Overexpression of miR-499-5p inhibited 2 μM DOX-induced apoptosis in cardiomyocytes. Cardiomyocytes were transfected with miR-499-5p mimic or negative control (NC) for 24 h and treated with 2 μM DOX for 24 h. Cell death was detected using the Tunel assay. Representative images show Tunel staining results (blue, DAPI; green, Tunel). Scale bar: 100 μm. **(E)** Statistical analysis of Tunel-positive cells in each group. Data are expressed as the means ± SD, *n* = 3 experiment ^∗^*P* < 0.05. **(F)** Knockdown of miR-499-5p sensitized cardiomyocytes to undergo apoptosis induced by DOX (0.2 μM). Cell death was detected using the Tunel assay. Data are expressed as the means ± SD; *n* = 3 experiment. ^∗^*P* < 0.05. **(G)** Overexpression of miR-499-5p inhibited DOX-induced mitochondrial fission in cardiomyocytes. Cells were transfected with a miR-499-5p mimic or negative control for 24 h. Representative images were captured using confocal microcopy. Scale bar: 2 μM. **(H)** Knockdown of miR-499-5p sensitized cardiomyocytes to undergo 0.2 μM DOX-induced cell death. Cell death was detected using the trypan blue assay. Data are expressed as the means ± SD, *n* = 3 experiment. ^∗^*P* < 0.05. **(I)** Overexpression of miR-499-5p inhibited 2 μM DOX-induced cell death in cardiomyocytes. Cell death was measured using trypan blue assays. Data are expressed as the means ± SD, *n* = 3 experiment. ^∗^*P* < 0.05. **(J)** Statistical analysis of the percentage of cells undergoing mitochondrial fission. Data are expressed as the means ± SD, *n* = 3 experiment. ^∗^*P* < 0.05. Cardiomyocytes were transfected with a miR-499-5p mimic or negative control for 24 h and treated with 2 μM DOX for 24 h. **(K)** Statistical analysis of the percentage of cells undergoing mitochondrial fission. Data are expressed as the means ± SD, *n* = 3 experiment. ^∗^*P* < 0.05. H9c2 cells were transfected with a miR-499-5p antagomiR or antagomiR control for 24 h and treated with 0.2 μM DOX for 24 h. The percentage of cells undergoing mitochondrial fission was determined.

miR-499-5p exerts a protective role in the pathogenesis of heart diseases and miR-499-5p mRNA levels are downregulated in cardiomyocytes during apoptotic stress and in the heart under pathological conditions (Matkovich et al., 2012). We detected miR-499-5p expression levels in cardiomyocytes exposed to DOX to investigate the role of miR-499-5p in DOX-induced cardiotoxicity. miR-499-5p expression was significantly downregulated after DOX (2 μM) treatment (Figure 1A). Cardiomyocytes were transfected with a miR-499-5p mimic. Real-time PCR demonstrated that miR-499-5p levels increased 4-fold compared to the negative control (Figure 1B). The miR-499-5p mimic efficiently inhibited mitochondrial fission (Figures 1G,J) and cell apoptosis (Figures 1D,E,I) in cardiomyocytes exposed to DOX (2 μM). We knocked down endogenous miR-499-5p using a miR-499-5p antagomiR to mimic the DOX-induced downregulation of miR-499-5p. Real-time PCR demonstrated that the miR-499-5p antagomiR efficiently knocked down the endogenous miR-499-5p (Figure 1C). Knockdown of miR-499-5p sensitized cardiomyocytes to DOX, which induced mitochondrial fission and apoptosis in cardiomyocytes at a lower concentration (0.2 μM) (Figures 1F,H,K). These results suggest the involvement of miR-499-5p in DOX cardiotoxicity and an attenuation of DOX-induced apoptosis in cardiomyocytes via inhibition of mitochondrial fission by miR-499-5p.

### miR-499-5p Attenuates Mitochondrial Fission and Apoptosis in DOX Cardiotoxicity *in vivo*

**FIGURE 2:**
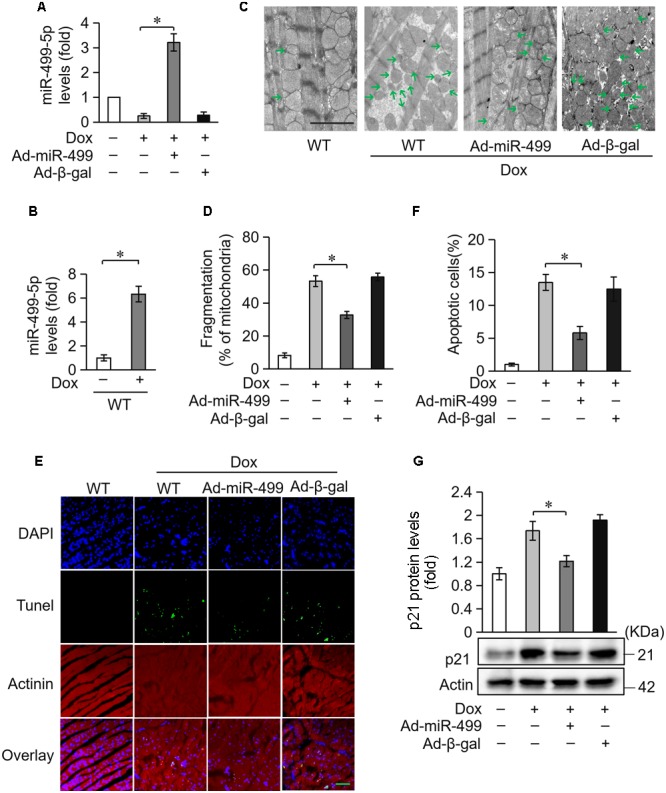
miR-499-5p attenuates mitochondrial fission and apoptosis *in vivo*. **(A)** miR-499-5p mRNA levels in adenovirus-harboring, miR-499-5p-overexpressing mice (Ad-miR-499) and the control mice administered DOX. *n* = 3 mice per group. Data are presented as the means ± SD, ^∗^*P* < 0.05. **(B)** Serum miR-499-5p expression was detected in the mice administered DOX by real-time PCR. *n* = 3 mice per group. Data are presented as the means ± SD, ^∗^*P* < 0.05. **(C,D)** Mitochondrial fission was analyzed using scanning electron microscope (SEM) in heart tissues from miR-499-5p-overexpressing mice and the control mice administered DOX. Scale bar, 2 μm. Arrows indicate mitochondria that underwent fission. **(C)** Statistical analyses of the percentage of mitochondrial fission. Mitochondrial fission was calculated as described in the Section “Materials and Methods.” Data are presented as the means ± SD, ^∗^*P* < 0.05; *n* = 5 mice per group. **(D,E)** Representative images revealed Tunel-positive cells in heart tissues. Green, Tunel-positive nuclei; blue, DAPI (4,6-diamidino-2-phenylindole)-stained nuclei; red, cardiomyocytes labeled with α-actinin antibody; scale bar, 20 μm. n = 5 mice per group. **(F)** Statistical analysis of Tunel-positive cells in each group. Data are presented as the means ± SD, ^∗^*P* < 0.05. **(G)** Western blotting shows the expression of p21. Actin served as a loading control.

We investigated the role of miR-499-5p in mice model to further validate its function in DOX-induced cardiotoxicity. miR-499-5p levels were significantly downregulated in DOX-treated mice heart (Figure 2A) while the serum miR-499-5p expression was significantly increased (Figure 2B). DOX administration induced abnormal mitochondrial fission and cell apoptosis in the mouse heart (Figures 2C–F). The adenovirus harboring miR-499-5p efficiently forced the expression of miR-499-5p in mouse heart (Figure 2A) and significantly attenuated cell mitochondrial fission and cell apoptosis in the heart following DOX administration (Figures 2C–F). Notably, we observed a significant increase in the protein levels of p21 in DOX-treated mice, which was inhibited in the miR-499-5p-overexpressing mice (Figure 2G). In conclusion, miR-499-5p attenuated DOX-induced mitochondrial fission and cell apoptosis in the mouse heart *in vivo*.

### miR-499-5p Attenuates DOX Cardiotoxicity in Mice

**FIGURE 3:**
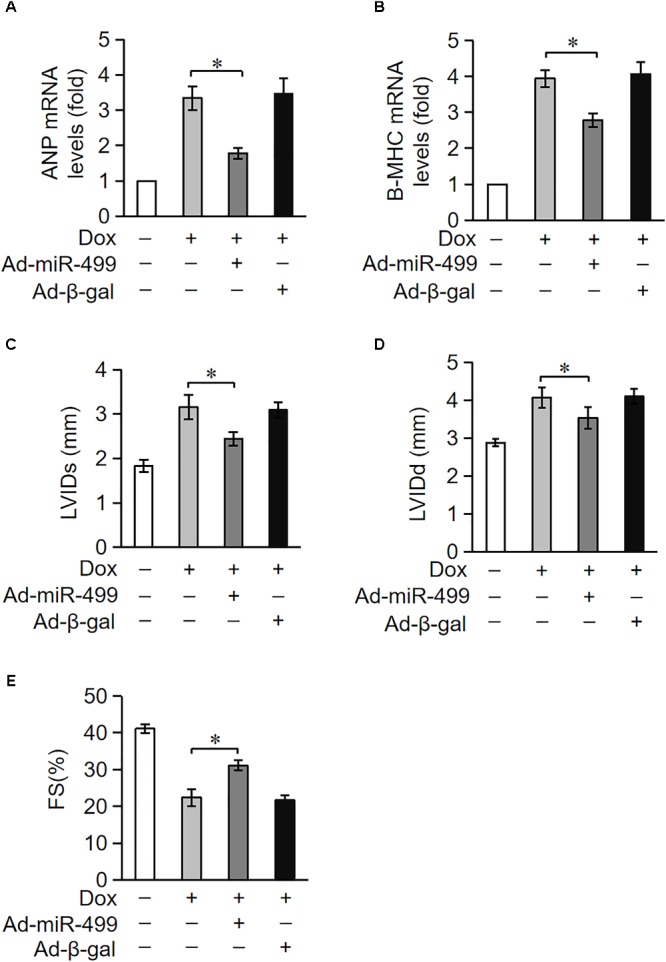
miR-499-5p attenuates DOX cardiotoxicity in mice. **(A–E)** miR-499-5p-overexpressing mice are resistant to left ventricular remodeling after DOX treatment. **(A,B)** The expression levels of ANP and β-myosin heavy chain (B-MHC) were detected using qRT-PCR. Data are expressed as the means ± SD, *n* = 3 experiment. ^∗^*P* < 0.05. **(C–E)** miR-499-5p-overexpressing mice or control mice were exposed to DOX or saline as described in the panel, and echocardiography was used to test heart function. LVIDs, systolic left ventricular internal diameter **(C)**; LVIDd, diastolic left ventricular internal diameter **(D)**; FS, fractional shortening of the left ventricular diameter **(E)**. Data are presented as the means ± SD, *n* = 8 mice per group. ^∗^*P* < 0.05.

Doxorubicin treatment caused a high degree of cardiomyocytes apoptosis. Because cardiomyocytes exhibit a limited ability to regenerate, cardiomyocytes loss during treatment cannot be replenished. Cardiomyocytes undergo compensatory hypertrophy, and the heart develops dilated cardiac hypertrophy with a decrease in heart function under the treatment of DOX. ANP and β-MHC are two biomarkers for the cardiac hypertrophy (Wang et al., 2015b). We measured the expression levels of ANP and β-MHC to examine the role of miR-499-5p in DOX-induced cardiac hypertrophy. ANP and β-MHC expression levels were significantly upregulated in mice administered DOX, but these increases were significantly inhibited in the miR-499-5p-overexpressing mice (Figures 3A,B). These results indicate that miR-499-5p inhibits the cardiac hypertrophy induced by DOX cardiotoxicity. We also examined the effect of miR-499-5p on cardiac remodeling in mice. Our results demonstrated that the cardiac remodeling was ameliorated in miR-499-5p-overexpressing mice as assessed using echocardiography (Figures 3C–E). In conclusion, miR-499-5p efficiently attenuated DOX cardiotoxicity in our mouse model.

### p21 Is a Target of miR-499-5p

**FIGURE 4:**
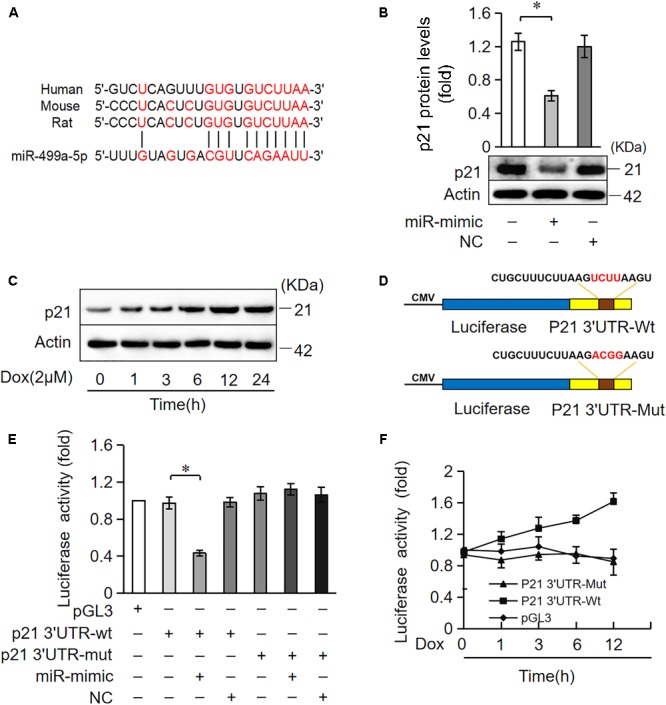
miR-499-5p participates in the regulation of p21 expression. **(A)** Analysis of the p21 3′UTR potential binding site of miR-499-5p using RNA hybrid. Potential complementary residues are shown in red. **(B)** Cardiomyocytes were transfected with a miR-499-5p mimic or negative control for 24 h. The expression levels of p21 were determined using Western blot. **(C)** Cardiomyocytes were treated with DOX (2 μM) at the indicated time points. The expression levels of p21 were detected using Western blots. **(D–F)** miR-499-5p directly targets the p21 3′UTR. **(D)** Schematic diagram of the reporter containing the putative miR-499-5p binding in the p21 3′UTR. **(E)** Luciferase activity detected in HEK-293 cells transfected with synthesized miR-499-5p mimic or negative control along with luciferase reporter constructs, as indicated. **(F)** Luciferase activity of the luciferase construct p21 3′UTR-Wt decreased with DOX treatment in cardiomyocytes. Data are expressed as the means ± SD, *n* = 3 except in panel; ^∗^*P* < 0.05.

miRNAs primarily target the 3′UTR region of target genes and negatively regulate target gene expression via the inhibition of translation or promotion of mRNA degradation (Bartel, 2009). We analyzed the potential targets of miR-499-5p using the bioinformatic program TargetScan to elucidate the molecular mechanisms by which it regulates cell apoptosis. miR-499-5p was predicted to bind to the 3′UTR region of p21, which is conserved in rats, mice and humans (Figure 4A). We also observed that p21 protein levels increased in cardiomyocytes treated with DOX in a time-dependent manner while miR-499-5p expression decreased, which suggests that p21 may be a target of miR-499-5p (Figures 1A, 4C). We forced the expression of miR-499-5p in cardiomyocytes to further analyze the regulatory role of miR-499-5p on p21. Overexpression of miR-499-5p significantly attenuated p21 expression in cardiomyocytes (Figure 4B). We used a luciferase assay system to examine whether miR-499-5p influenced p21 expression via a direct targeting of p21 3′UTR. We cloned p21 3′UTR containing the miR-499-5p binding site downstream of the luciferase reporter gene, p21 3′UTR-Wt, to examine luciferase activity driven by the 3′UTR of p21. We also generated a mutated luciferase construct, p21 3′UTR-Mut, with mutations introduced into the miR-499-5p-binding site of p21 3′UTR (Figure 4D). Both the wild-type 3′UTR of p21 (p21 3′UTR-Wt) and the mutated 3′UTR of the p21 (p21 3′UTR-Mut) showed a similar luciferase activity with the luciferase reporter gene (pGL3) (Figure 4E). However, the wild-type 3′UTR of p21 exhibited low luciferase activity in the presence of miR-499-5p while the mutated 3′UTR did not produce a significant response to miR-499-5p (Figure 4E). A luciferase activity assay in cardiomyocytes also demonstrated that DOX (2 μM) treatment increased the luciferase activity regulated by wild-type p21 3′UTR. The mutation of miR-499-5p-binding site slowed the increase in these luciferase activity (Figure 4F). Taken together, these results suggest that p21 is a specific target of miR-499-5p.

### p21 Attenuates Mitochondrial Fission and Apoptosis in Cardiomyocytes Treated With DOX

**FIGURE 5:**
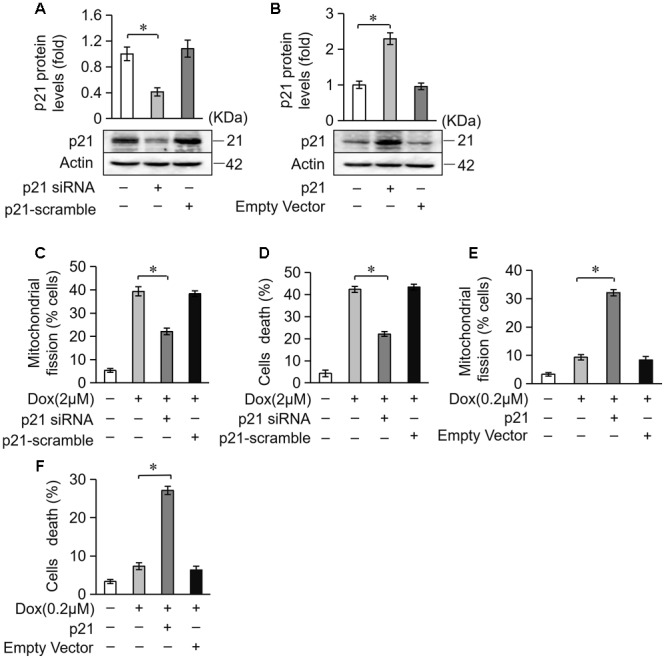
p21 attenuates mitochondrial fission and cell death in H9c2 cells treated with DOX. **(A)** The expression levels of p21 were detected using Western blot in cardiomyocytes transfected with p21 siRNA. **(B)** The expression levels of p21 were detected using Western blot in cardiomyocytes transfected with a p21-overexpressing vector. Actin served as the loading control. **(C,D)** Knockdown of p21 inhibited 2 μM DOX-induced mitochondrial fission and cell death. Cardiomyocytes were transfected with p21 siRNA or p21 scramble for 24 h and treated with DOX (2 μM) for 24 h. **(E,F)** Overexpression of p21 increased mitochondrial fission and cell death induced by low-dose DOX (0.2 μM). Cardiomyocytes were transfected with p21 or empty vector for 24 h and treated with 0.2 μM DOX for 24 h. ^∗^*P* < 0.05; Data are expressed as the means ± SD, *n* = 3 experiment. ^∗^*P* < 0.05.

Next, we investigated the potential role of p21 in DOX-induced cardiotoxicity. We knocked down endogenous p21 using siRNA (Figure 5A). Knockdown of endogenous p21 significantly decreased mitochondrial fission and cell death in cardiomyocytes exposed to DOX (2 μM) (Figures 5C,D). We also overexpressed p21 using the pcDNA3.1 eukaryotic expression vector with a CMV promoter (Figure 5B). p21 overexpression induced massive mitochondrial fission and sensitized cardiomyocytes to undergo apoptosis under a lower concentration of DOX (0.2 μM) (Figures 5E,F). Taken together, these results suggest that p21 promotes mitochondrial fission program and cell apoptosis in cardiomyocytes exposed to DOX.

### miR-499-5p Attenuates Mitochondrial Fission and Apoptosis via Targeting p21

**FIGURE 6:**
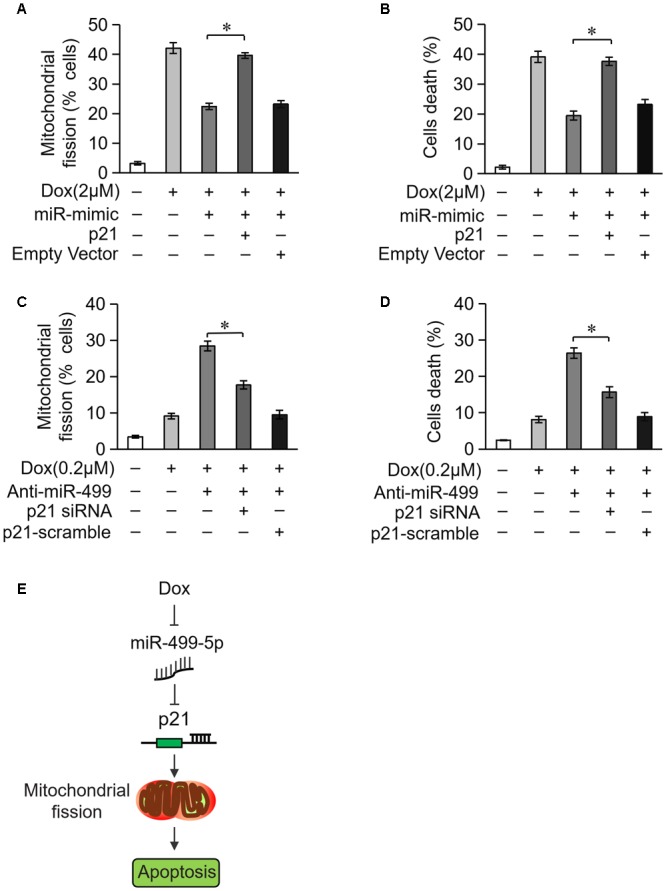
miR-499-5p attenuates mitochondrial fission and apoptosis via targeting p21. **(A,B)** miR-499-5p attenuated the sensitivity of cardiomyocytes to DOX treatment, which was abolished by p21 overexpression. Statistical analysis of the mitochondrial fission **(A)** and cell death **(B)** in cardiomyocytes following DOX (2 μM) treatment. **(C,D)** Knockdown of miR-499-5p increased the sensitivity of cardiomyocytes to DOX (0.2 μM) treatment, which was abolished by p21 knockdown. Statistical analysis of the mitochondrial fission **(C)** and cell death **(D)** in cardiomyocytes following DOX (0.2 μM) treatment. **(E)** Schematic diagram of this study. Data are presented as the means ± SD, *n* = 3 experiment. ^∗^*P* < 0.05.

We examined whether miR-499-5p attenuated mitochondrial fission and cell apoptosis via the targeting of p21. Overexpression of miR-499-5p inhibited DOX-induced mitochondrial fission and cell death while forced expression of p21 attenuated this inhibitory effect on mitochondrial fission (Figure 6A) and cell death (Figure 6B). Knockdown of the endogenous miR-499-5p sensitized cardiomyocytes to undergo mitochondrial fission and cell death at a lower dose of DOX (0.2 μM) while simultaneously knockdown of p21 inhibited these mitochondrial fission (Figure 6C) and cell death (Figure 6D). These data suggest that miR-499-5p attenuates DOX-induced mitochondrial fission and apoptosis in cardiomyocytes via the targeting of p21.

### miR-499-5p Is Not Involved in DOX-Induced Apoptosis in Cancer Cells

**FIGURE 7:**
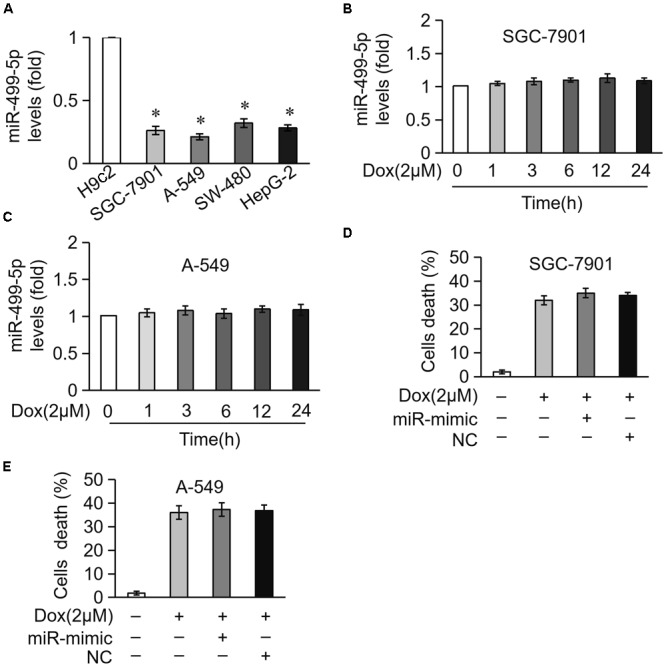
miR-499-5p is not involved in DOX-induced apoptosis in cancer cells. **(A)** Real-time PCR results show the miR-499-5p expression levels in H9c2, SGC-7901, A-549, SW-480, and HepG-2 cells. ^∗^*P* < 0.05 versus cardiomyocytes. **(B,C)** Real-time PCR results show miR-499-5p levels in SG-7901 **(B)** and A-549 **(C)** cells treated with DOX (2 μM) for the indicated times. **(D,E)** Statistical analysis of the cell death in SG-7901 **(D)** and A-549 **(E)** cells transfected with miR-499-5p mimic following treatment with DOX (2 μM). Data are expressed as the means ± SD, *n* = 3 experiment.

Our results showed that miR-499-5p is a promising factor for preventing DOX cardiotoxicity in cancer therapy, but it is important to exclude its role in the apoptosis of tumor cells. We compared differences in miR-499-5p expression levels between cardiomyocytes and tumor cells. The results revealed that miR-499-5p was expressed at very low levels in tumor cells, including SGC-7901, A-549, SW-480 and HepG-2s, compared to cardiomyocytes (Figure 7A). We examined miR-499-5p expression levels during cell apoptosis in tumor cells treated with DOX. The results demonstrated that miR-499-5p expression was insensitive to DOX treatment in tumor cells (Figures 7B,C). Notably, miR-499-5p overexpression did not affect DOX-induced cell death in tumor cells (Figures 7D,E). These results indicate that miR-499-5p is not involved in the process of DOX-induced tumor cell apoptosis.

## Discussion

Doxorubicin is a powerful drug in the clinical fight against cancer. However, its cardiotoxicity is a major challenge and limits its application. A total of 20% of patients who receive DOX treatment develop heart failure, and DOX accumulation reaches 500 mg/m^2^ in these patients’ heart (Chatterjee et al., 2010). An understanding of the molecular mechanisms will ameliorate the cardiotoxicity and increase the clinical efficacy of DOX. miRNAs play important roles in DOX-induced cardiotoxicity. miR-146a is involved in DOX-induced cardiomyocyte cell death via the targeting of ErbB4 (Horie et al., 2010). miR-30 targets GATA to participate in DOX-induced cardiomyocyte apoptosis (Roca-Alonso et al., 2015). MicroRNA-208 also contributes to DOX-induced cardiomyocyte cell death via the targeting of GATA4 (Tony et al., 2015). Our previous work found that miR-532-3p was involved in DOX-induced cardiotoxicity via the repression of ARC expression (Wang et al., 2015b). miR-140-5p directly targets Nrf2 and Sirt2 and increases DOX-induced oxidative damage via altering FOXO3a expression levels (Zhao et al., 2018). We reported here that miR-499-5p efficiently prevented DOX cardiotoxicity via attenuating mitochondrial fission and cell apoptosis and targeting of p21 (Figure 6E).

Two other types of non-coding RNAs, lncRNAs and circRNAs, garnered great attention recently, and these non-coding RNAs are also involved in DOX cardiotoxicity and myocardial damage. lincRNA-p21 participates in DOX-related cardiac cell senescence via regulation of the Wnt/beta-catenin signaling pathway, and silencing of lincRNA-p21 effectively protects against DOX cardiotoxicity (Xie et al., 2018). Qki (Quaking), which is a RNA binding protein, inhibits DOX cardiotoxicity via regulation of cardiac circular RNAs (Gupta et al., 2018). Knockdown of MiRt1 improves cardiac function, reduces apoptosis of cardiomyocytes and attenuates inflammatory cell infiltration *in vivo* (Li et al., 2017). lncRNA-cardiac autophagy inhibitory factor (CAIF) inhibits autophagy and attenuates myocardial infarction via the targeting of p53-mediated transcription of myocardin (Liu C.Y. et al., 2018). Microarray analysis found a total of 63 differentially expressed circRNAs, including 29 upregulated and 34 downregulated circRNAs, during myocardial infarction (Wu et al., 2016). Mitochondrial fission and apoptosis-related circRNA (MFACR) regulates mitochondrial fission and apoptosis in the heart via direct targeting of miR-652-3p (Li et al., 2018). In conclusion, lncRNAs and circRNAs are potential targets for the prevention of DOX cardiotoxicity. Further study is needed to elucidate the functional role of these two non-coding RNAs.

The role of miR-499-5p in certain types of tumors is controversial. Several studies demonstrated that miR-499-5p suppressed cancer development and progression. Overexpression of miR-499-5p downregulated ETS1 expression, and it inhibited the migration and infiltration of HepG-2 cells (Wei et al., 2012). miR-499-5p could also inhibit the growth of tumor cells and improves the effectiveness of cancer treatment (Ando et al., 2014). miR-499-5p may also act as a tumor suppressor gene via the targeting of VAV3 (Li M. et al., 2016). However, other studies revealed that miR-499-5p was abnormally expressed in non-small cell lung cancer tissues, and it may be used as a biomarker for early diagnosis and evaluation of patient prognosis (Li et al., 2014). Our study found that miR-499-5p produced no effect on tumor cells, including gastric cancer, non-small cell lung cancer, colon cancer or liver cancer, which suggests that it increases the therapeutic potential of the tumor and protects the heart without promoting tumor development. Therefore, miR-499-5p may be an effective factor in the prevention of DOX cardiotoxicity.

Single nucleotide polymorphisms (SNPs) are the most frequent type of variation in the genome. Numerous study demonstrated that functional SNPs in miRNA genes affect different signaling pathways via altering the expression levels of target genes (Zhi et al., 2012; Chen et al., 2014; Liu et al., 2017). Increasing evidence suggests that miRNA polymorphisms are associated with the susceptibility to heart diseases, such as myocardial infarction and coronary heart disease. Previous studies reported that miR-196a2 rs11614913 (Xu et al., 2009; Zhi et al., 2012), miR-146a rs2910164 (Chen et al., 2014), and miR-149 rs71428439 (Ding et al., 2013) were associated with the risk of heart disease. miR-499-5p is a heart-rich miRNA under physiological conditions, and some studies demonstrated that miR-499-5p levels were downregulated under pathological conditions (van Rooij et al., 2009; Wang et al., 2011). The plasma level of miR-499-5p may help distinguish acute myocardial infarction and heart disease in patients (Olivieri et al., 2013). Our previous study found that the SNP rs3746444 of the miR-499 precursor affected the expression level and anti-apoptotic function of miR-499-5p (Ding et al., 2018). Whether SNPs in miRNA-499-5p are related to the susceptibility to cardiotoxicity need to be further investigated, which is of great significance for guiding medication and overcoming drug cardiotoxicity.

Well-balanced mitochondrial dynamics play an important role in cell life (Archer, 2014). Abnormal mitochondrial fission leads to the onset of cardiomyocyte apoptosis and the development of heart failure (Lee et al., 2004; Ong and Hausenloy, 2010). DOX-induced mitochondrial dysfunction is currently the major cause of cardiotoxicity (Ichikawa et al., 2014), and it was also documented in heart failure patients (Lebrecht et al., 2005). Our present study revealed the pivotal role of p21 in the promotion of mitochondrial fission caused by DOX treatment. Drp1-mediated mitochondrial fission is an important component of cell apoptosis (Martinou and Youle, 2006), and p21 participates in the Drp1-mediated mitochondrial fission that promotes HCC cell proliferation (Zhan et al., 2016). p21 also participated in the p53-mediated mitochondrial apoptosis program in nickel (II)-induced nasal epithelial cytotoxicity (Lee et al., 2016). However, whether p21 regulates DOX-induced mitochondrial fission via Drp1 or p53 need to be further examined.

Taken together, we report that the downregulation of miR-499-5p in cardiomyocytes exposed to DOX is involved in DOX cardiotoxicity. There is emerging evidence for the involvement of p21 in the promotion of mitochondrial fission and cell apoptosis in cardiomyocyte exposed to DOX. miR-499-5p prevented mitochondrial fission and cell apoptosis in cardiomyocytes exposed to DOX via the targeting of p21 (Figure 6E). Therefore, the development of new therapeutic strategies based on the miR-499-5p-p21 axis is promising for the overcoming of DOX cardiotoxicity in cancer treatment.

## Ethics Statement

This study was carried out in accordance with the recommendations of Institutional Animal Care and Use Committee of Qingdao University Medical College. The protocol was approved by Institutional Animal Care and Use Committee of Qingdao University Medical College.

## Author Contributions

JW and TX designed the research. QW and WD performed the cellular experiments. XZ and TX performed the animal experiments. TY and XJ constructed the reporter construct and adenoviruses. WY and ZL analyzed the cardiac function. QW and JW wrote the manuscript. All authors approved the final version of the manuscript.

## Conflict of Interest Statement

The authors declare that the research was conducted in the absence of any commercial or financial relationships that could be construed as a potential conflict of interest.
